# Double and Triple Differential Cross Sections for Single Ionization of Benzene by Electron Impact

**DOI:** 10.3390/ijms22094601

**Published:** 2021-04-27

**Authors:** Ana I. Lozano, Filipe Costa, Xueguang Ren, Alexander Dorn, Lidia Álvarez, Francisco Blanco, Paulo Limão-Vieira, Gustavo García

**Affiliations:** 1 Instituto de Física Fundamental, Consejo Superior de Investigaciones Científicas, Serrano 113-bis, 28006 Madrid, Spain; filipe2626@gmail.com (F.C.); lid.alvarez@iff.csic.es (L.Á.); 2 Atomic and Molecular Collisions Laboratory, CEFITEC, Department of Physics, Universidade NOVA de Lisboa, 2829-516 Caparica, Portugal; plimaovieira@fct.unl.pt; 3 Max Planck Institute for Nuclear Physics, 69117 Heidelberg, Germany; ren@mpi-hd.mpg.de (X.R.); A.Dorn@mpi-k.de (A.D.); 4 Departamento de Estructura de la Materia Física Térmica y Electrónica e IPARCOS, Universidad Complutense de Madrid, Plaza de Ciencias 1, 28040 Madrid, Spain; pacobr@fis.ucm.es

**Keywords:** electron scattering cross sections, benzene ionization

## Abstract

Experimental results for the electron impact ionization of benzene, providing double (DDCS) and triple differential cross sections (TDCS) at the incident energy of 90 eV, measured with a multi-particle momentum spectrometer, are reported in this paper. The most intense ionization channel is assigned to the parent ion (C_6_H_6_^+^) formation. The DDCS values are presented for three different transferred energies, namely 30, 40 and 50 eV. The present TDCS are given for two fixed values of the ejected electron energy (E_2_), at 5 and 10 eV, and an electron scattering angle (θ_1_) of 10°. Different features related to the molecular orbitals of benzene from where the electron is extracted are observed. In addition, a semi-empirical formula to be used as the inelastic angular distribution function in electron transport simulations has been derived from the present DDCS result and compared with other expressions available in the literature.

## 1. Introduction

Ionizing radiation transfers energy along its path within a biological system being closely related to linear energy transfer (LET). As a consequence of local photoelectric and Compton effects, a considerable number of low-energy electrons (LEE) are produced as final products of the radiation-matter interactions [1]. Nowadays, it is well-established that those electrons may significantly alter the local chemistry and potentiate strong biochemical changes in the medium, leading to biological damage, yielding single and double DNA strand breaks via dissociative electron attachment resonances [2,3] and non-resonant processes as ionization [4], among others. The characterization of the damage induced by LEE at the molecular level requires detailed knowledge of the interaction probabilities [5] (i.e., the cross sections), which provide relevant input information for the Monte Carlo simulation procedures used in radiotherapy [6]. Despite considerable progress in this area in recent decades from both experimental and theoretical methodologies, the study of several molecules becomes harder, or even impossible, as the size of the molecule increases. For this reason, electron interactions with model molecules are being extensively investigated, to serve as a benchmark for testing the validity of electron scattering of more complex molecules.

Benzene (C_6_H_6_) is the simplest aromatic hydrocarbon, and therefore acts as a reference model for understanding the physico-chemical properties of a vast set of biologically relevant molecules. Nevertheless, although one finds in the literature [7,8,9,10,11] (and references therein) several studies on electron interactions with benzene, detailed investigations of the electron-impact ionization dynamics have not been performed to date, as far as the authors are aware. Nonetheless, different studies on the ionization and fragmentation dynamics of benzene produced by intense laser fields [12,13,14] or excited metastable atoms [15] can be found in the literature. On the other hand, different studies on the electron-impact ionization dynamics for other complex biologically relevant molecules such as DNA bases or pyrimidine are also reported [16,17,18,19,20].

The main goals of this study are twofold. Firstly, we aim to provide an experimental characterization of the electron-impact ionization dynamics of benzene at relatively low-electron energy (90 eV). For that, kinematically complete experiments, (*e*,*e*) and (*e*,*2e*) studies, were performed with a reaction microscope apparatus [21,22]. In this experiment, one can simultaneous probe energy and angular distributions for all the particles involved in the ionization process (scattered electron, secondary ejected electron and recoil ion), providing double and triple differential cross section (DDCS and TDCS) values. Secondly, through the obtained experimental results, we seek to check the accuracy of a semi-empirical formula [23] to reproduce the inelastic angular distribution of scattered electrons, as a function of that for the elastic scattering and the energy transferred during the collision.

The remainder of this paper comprises three parts. The obtained experimental results are presented and discussed in the next part. In addition, the accuracy of the semi-empirical formula is checked, and some improvements are proposed. In the following section, the experimental setup is described. Finally, the main findings are summarized, and some conclusions are drawn.

## 2. Results and Discussion

The outermost molecular orbitals (MOs) of benzene in terms of D_6h_ symmetry in its ground state (^1^A_1g_) are tabulated in Table 1 [24], together with their binding energies [24], i.e., ionization energy (IE), as well as with their respective ionization cross sections at 90 eV electron impact energy obtained by means of the binary-encounter-Bethe (BEB) method [25].

In the present experiment, the most intense ionization channel was found to be the parent ion (C_6_H_6_^+^), whereas the signal for H-loss (C_6_H_5_^+^) was difficult to discern from the contribution of other C_6_H_n_^+^ fragments, so we focused on the investigation of C_6_H_6_^+^ only.

### 2.1. Double Differential Ionization Cross Sections (DDCS)

The single ionization of a target by an incoming electron produces two outgoing electrons, together with the residual ion. DDCS, ($d^{3}\sigma /{d\Omega }_{1}{\mathrm{dE}}_{1}$), would be obtained by measuring the energy and angular distribution of one outgoing electron. Therefore, DDCS are differential both in energy and angle.

**Figure 1:**
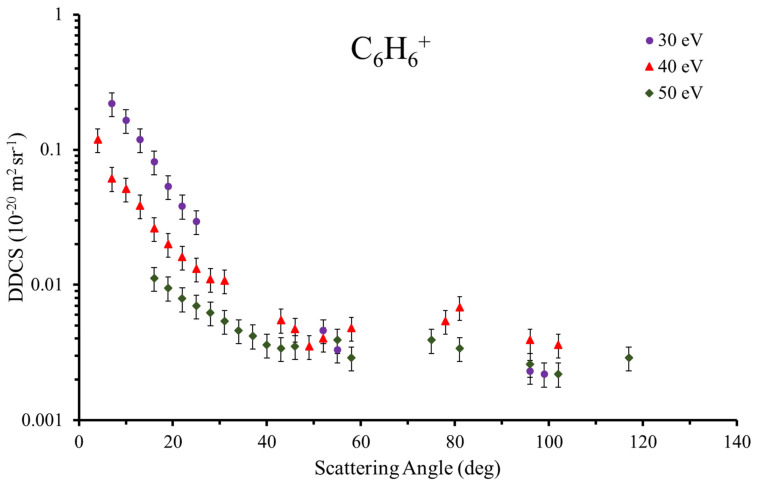
Experimental absolute double differential cross sections (DDCS) for producing the ion parent of benzene (C_6_H_6_^+^) at 90 eV of impact energy for different transferred energies (30, 40, and 50 eV).

The experimental results for DDCS to ionize benzene at an impact energy of 90 eV (Figure 1) are summarized in Table 2, together with their estimated uncertainties (δ). The data were obtained by analyzing the angular distribution of the scattered electrons as a function of their energies, i.e., the energy transferred to the target from the recorded 2D surfaces. Here, we present cuts through the two-dimensional (2D) DDCS surfaces for three transferred energies (ΔE), namely 30, 40 and 50 eV. The absolute values were assigned from normalization with those calculated for the ionization of He atoms [26], by applying the well-established convergent close-coupling method. As the He to benzene density ratios, which are necessary to normalize the results, were determined using the total ion yields as well as the total ionization cross sections, we conservatively consider an uncertainty associated with the absolute values of about 20%.

A close inspection of the DDCS as a function of the transferred energy in Figure 1 reveals that, below 40°, the cross section increases, with the decreasing scattering angle being more pronounced as the transferred energy decreases. In contrast, above 40°, an almost isotropic behavior is observed, regardless of the transferred energy. A similar behavior was recently observed in our experimental results of DDCS for the production of the pyridine parent ion (C_6_H_5_N^+^) [20]. Yet, the cross-section magnitude to produce the parent ion is slightly higher for the case of the benzene molecule.

### 2.2. Triple Differential Ionization Cross Sections (TDCS)

**Figure 2:**
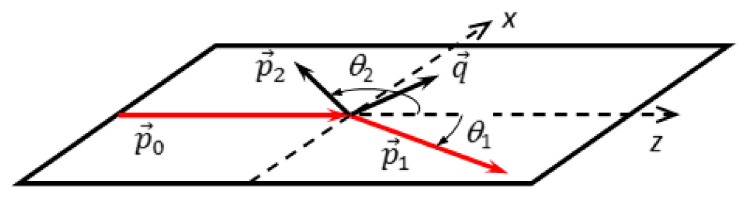
The coplanar scattering geometry. For details see text.

As mentioned above, an ionizing collision produces an electron pair of one scattered and one ejected electron in addition to the residual ion. The ionization kinematics is completely determined if in addition to the known projectile momentum, e.g., the emission angles of both outgoing electrons and the energy of one of the electrons are measured. The respective TDCS, ($d^{3}\sigma /{d\Omega }_{1}{d\Omega }_{2}{\mathrm{dE}}_{2}$), therefore, is fully differential. Figure 2 shows the scattering geometry considered in the present work which includes the incoming projectile with momentum ${\vec{p}}_{0}$ and the two outgoing electrons with momenta ${\vec{p}}_{1}$ and ${\vec{p}}_{2}$ as well as emission angles *θ*_1_ and *θ*_2_ with respect to the projectile forward direction. It is most likely that the energy sharing among the electrons is asymmetric, with one faster electron (${\vec{p}}_{1}$) which is preferentially emitted to the forward direction and which can be well identified with the scattered projectile. Then, the momentum transferred by the projectile to the target is given by $\vec{q}={\vec{p}}_{0}-{\vec{p}}_{1}$. Generally, the TDCS can be obtained for the emission of the ionized electron into the full solid angle and is represented, e.g., as a 3D surface [27,28]. In this study, we only consider a cut of the 3D surface, in particular through the projectile scattering plane (xz) (see ref. [29] for further details). In the following, the TDCS is presented for the fixed projectile scattering angle *θ*_1_ and particular energies E_2_ of the ionized electron, as a function of its emission angle *θ*_2_ within the plane.

**Figure 3:**
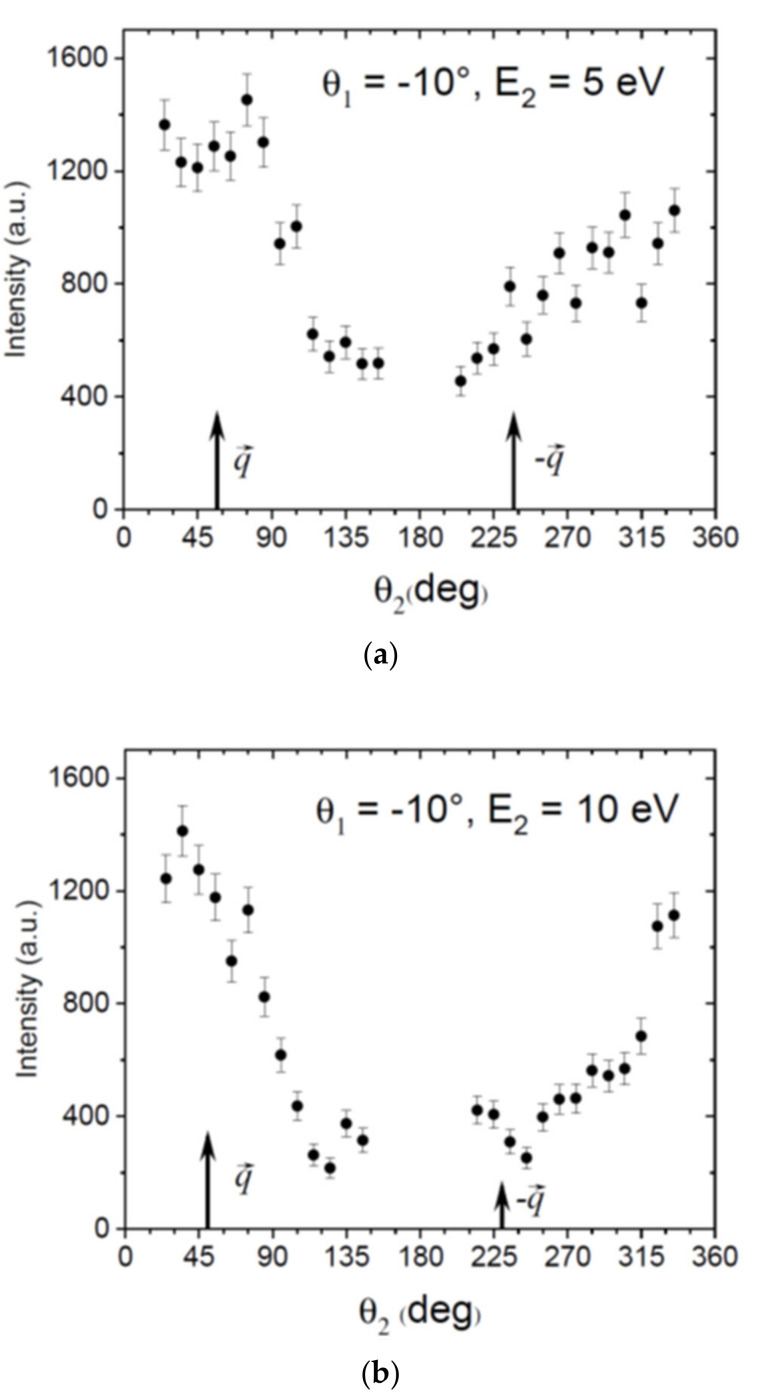
Experimental relative TDCS (in arbitrary units) for producing the parent ion of benzene (C_6_H_6_^+^) at 90 eV electron impact energy as a function of the ejected electron emission angle *θ*_2_. The scattered projectile angle is fixed to *θ*_1_ = −10° while the ejected electron energy E_2_ is chosen to be 5 eV (**a**) and 10 eV (**b**). The experimental results are for the scattering plane (xz). The angles of the momentum transfer vector $\vec{q}$ and its opposite $-\vec{q}$ are indicated by arrows.

Figure 3 shows the experimental TDCS in the xz-scattering plane where the values are not on an absolute scale. The panels (a) and (b) are for ejected electron energies E_2_ = 5 and 10 eV, respectively, and the scattered electron angle is *θ*_1_ = −10°. The direction of the momentum $\vec{q}$ transferred by the projectile to the molecular target and its opposite direction $-\vec{q}$ are indicated by arrows. In the same way as the DDCS, these data were recorded in coincidence with the parent ion and, therefore, for the ionization of the highest three valence orbitals 1e_1g_, 3e_2g_ and 1a_2u_. Normally, atomic and molecular TDCS show two maxima, one for electron emission roughly along $\vec{q}$ and one for −$\vec{q}$. The first can be assigned to a binary collision of the projectile and the bound target electron and is named binary peak. The second involves additional backscattering of the ionized electron in the potential of the residual ion and is called recoil peak. In Figure 2, we see pronounced and broad binary peaks approximately along $\vec{q}$ extending to *θ*_2_ = 0° and beyond. Interestingly, the regularly observed angular shift away from $\vec{q}$ to larger angles due to the so-called post collision interaction (PCI), which is the repulsion of the two outgoing electrons, is not observed. In contrast, the binary peaks are slightly shifted towards the forward direction. The binary peak width and shape are determined by the collision dynamics, but also by the bound orbital momentum profile. This is because for ionization in a binary collision, the ejected electron carries the initial bound state momentum to which the momentum transferred in the collision is added. The uppermost three valence orbitals of benzene have *π*-character with amplitude minima at zero momentum [30]. As a result, a minimum in the cross section along $\vec{q}$ is also expected for the so-called Bethe ridge condition where the absolute value of the ejected electron momentum *p*_2_ is close to the magnitude of the momentum transfer *q*. Here, this is the case for E_2_ = 5 eV, where *p*_2_ = 0.61 a.u., which must be compared to *q* = 0.5 a.u. At E_2_ = 10 eV, the respective electron momentum is *p*_2_ = 0.86 a.u. and, thus, more different from *q.* As a consequence, no minimum is found in the binary peak. In the $-\vec{q}$ direction, no clear recoil peak can be identified. Nevertheless, there is a finite cross section in this angular region which is lower for E_2_ = 10 eV. For this energy, there is an indication of a small recoil peak at about 180° according to the wings observed around 150° and 210°, while its center is in the angular region, which is not covered by the spectrometer.

It is interesting to compare these data with results from other cyclic molecules. Recent results from tetrahydrofuran (THF) at similar kinematics [31] show a much more isotropic and less structured TDCS, which is typical and regularly observed for larger and relatively complex molecules [32,33]. In these cases, this was tentatively assigned to strong scattering of the ionized electron in the multi-center potential of the residual ion. Here, aromatic rings seem to behave differently; in addition to the present results for benzene, for phenol at a higher impact energy, a rather distinct binary peak was found accompanied with rather small intensity in the recoil region [34]. This could be explained by the strong delocalization of the highest *π*-orbitals in the benzene aromatic ring, which results in a comparatively narrow momentum distribution of bound orbitals, and possibly also by a reduced scattering of the ejected electron in the ionic potential. In this case, further, more systematic and detailed experimental studies including a theoretical treatment are required.

### 2.3. Semi-Empirical Formula

Input information required for event-by-event Monte Carlo procedures include inelastic angular distribution functions. Apart from the growing efforts to obtain accurate inelastic differential cross sections either from theoretical or experimental methods, Monte Carlo simulations often rely on approximate equations. With the main objective being to find an accurate approximation to comprehensively describe the interaction dynamics, a semi-empirical formula (Equation (1)) was derived from differential energy loss spectra, as thoroughly explained in Ref. [30]. This equation approximates the inelastic scattering angular distribution to that of the elastic contribution by including a correction exponent which depends on the incident and transferred energies [30]. The efficiency of this empirical equation has been proven in different electron transport simulations for an extensive set of biological relevant molecules [5,20,23,35].

**Figure 4:**
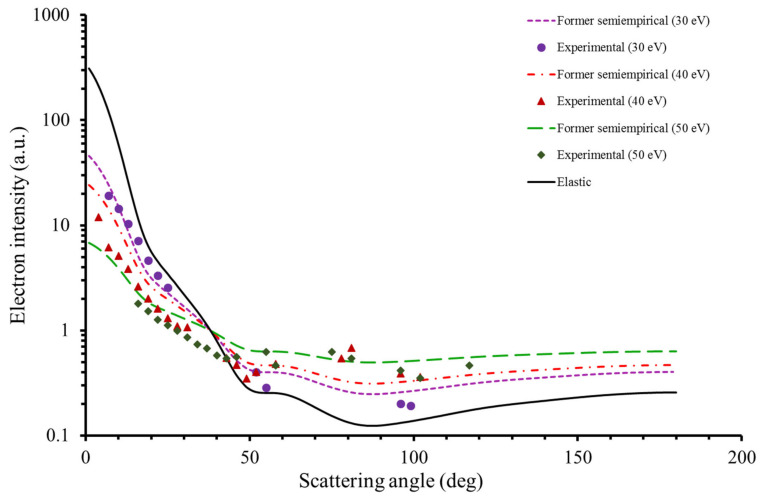
Inelastic angular distribution obtained from our former semi-empirical formula (Equation (1)) together with the experimental DDCS for transferred energies of 30, 40 and 50 eV and impact energy of 90 eV. Note that the elastic distribution is also included.

Figure 4 shows the experimental DDCS results together with the angular distribution given by the elastic scattering, as calculated with our IAM-SCAR+I [36,37] procedure, and that derived from the empirical Equation (1). As shown in this figure, Equation (1) reasonably accurately reproduces the experimental data for transferred energies of about 30 eV. However, for ∆E > 30 eV, especially for scattering angles below 40°, Equation (1) does not give a good description of the scattering dynamics, overestimating the experimental findings. A similar behavior has been observed for the case of pyridine [20].

(1) $$\frac{d^{2}\sigma \left(E\right)}{d\Omega d\Delta E}\propto {\left(\frac{d\sigma \left(E\right)}{d\Omega }\right)}_{\mathrm{el}}^{1-\Delta E/E}$$

In order to accurately reproduce the experimental results for transferred energies above 30 eV, we propose an alternative equation which is based on the present experimental DDCS results and is shown in (Equation (2)).

(2) $$\frac{d^{2}\sigma \left(E\right)}{d\Omega d\Delta E}\propto \;{\left(\frac{d\sigma \left(E\right)}{d\Omega }\right)}_{\mathrm{el}}^{{\left(1-\Delta E/E\right)}^{\alpha }}$$

**Figure 5:**
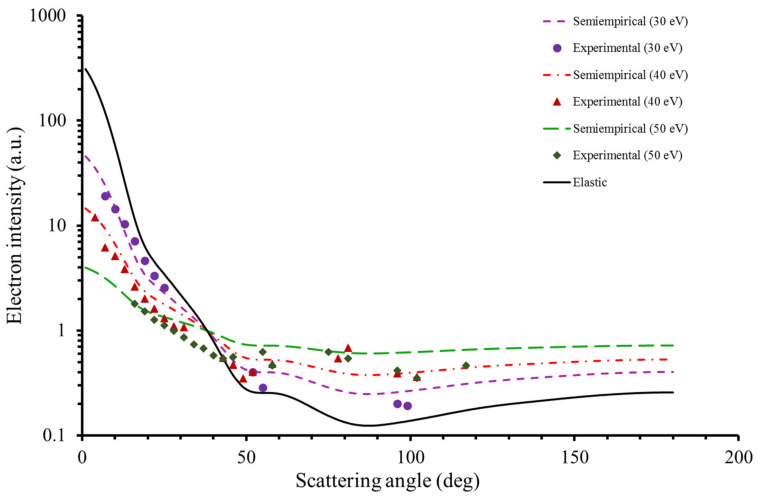
Same as in Figure 3 but now using the new adopted Equation (2). See text for details.

This new approximation results from applying an exponent α to the $\left(1-\Delta E/E\right)$ term, where α takes the value of 1.3, according to the present experimental data, as well as to those previously obtained for pyridine [20]. As seen in Figure 5, the resulting angular distribution dependence obtained with Equation (2) more accurately reproduces the experimental data for all the transferred energies considered in this study.

## 3. Experimental Setup

**Figure 6:**
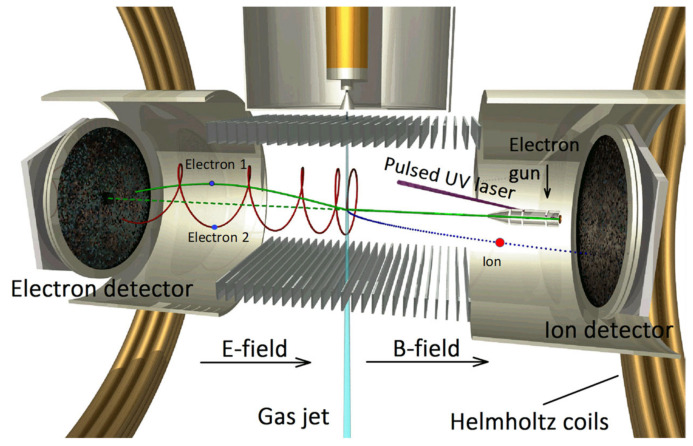
Schematic diagram of the reaction microscope used to analyze the angular and energy resolved double and triple differential cross sections, for electron induced ion fragmentation to benzene molecules.

The experimental setup used to investigate DDCS and TDCS is a spectrometer of multicoincidence multielectron recoil-ion momentum (reaction microscope), which was specifically designed for electron-impact ionization studies (Figure 6). This apparatus has been described extensively elsewhere [21,22]. In brief, a well-focused pulsed electron beam of about 0.5 ns duration, 0.5 eV energy spread, and 40 kHz pulse repetition frequency is crossed with a cold target beam created by supersonic expansion. The benzene expands through different stages and is admitted into the main scattering chamber where the background pressure is about 10^−8^ mbar. The scattered and the ejected electrons and the positive recoil ion, produced in the ionizing collisions, are extracted by parallel electric and magnetic fields, which are then projected into two position- and time-sensitive detectors in opposite directions. Triple-coincidences of both outgoing electrons and the benzene cation were recorded (*e*, *2e* + *ion*), therefore providing the necessary information to derive DDCS and TDCS. From the positions of the hits on the detector and the corresponding time-of-flight measurements, the momentum vectors of the final state particles can be determined. The full solid angle is almost entirely covered with this configuration, in particular 100% of the recoil ions and about 80% of secondary electrons with energies below 15 eV are detected. The projectile beam axis is adjusted to be exactly parallel to the electric and magnetic extraction field directions. Because of this alignment, after crossing the target gas jet, the projectile beam reaches the center of the electron detector where a central hole in the micro-channel plates allows the beam to pass undetected. This hole causes a blind angular range for electrons with small forward and backward emission angles which depends on the electron energy. Some secondary electrons, which undergo an integer number of cyclotron revolutions in the magnetic field, can also hit the mentioned hole. These events require several experimental runs, with different extraction conditions, in order to maximize the accessible angular range. For this purpose, two different electric extraction fields were applied during the measurements. Data for coincidence analysis were accumulated simultaneously for all covered scattering angles (*θ*_1_) and the respective scattered and ejected electron energies (E_1_ for DDCS and E_2_ for TDCS). With this procedure, the obtained DDCS and TDCS values were internormalized. The electron energy was calibrated using the ionization energy of neon, which was measured in the same experimental setup. The angular and energy resolution of this experimental system for the impact energy studied lie at around 2° and 2 eV, respectively.

## 4. Conclusions

The present study constitutes the first kinematically complete experiment on the electron-impact ionization dynamics of benzene. Novel results of DDCS and TDCS for this highly relevant molecule have been reported for the parent ion (C_6_H_6_^+^) at 90 eV impact energy. The DDCS have been obtained for three different transferred energies, showing that below 40°, the cross section continues to get more pronounced as the transferred energy decreases. As far as TDCS are concerned, experimental results in the coplanar xz-plane have been shown for two fixed values of the ejected electron energy E_2_ and the scattered electron angle *θ*_1_ = −10°. The TDCS show pronounced binary peaks with indications of a central minimum at Bethe ridge kinematics. The binary peak angular positions are shifted away from the momentum transfer and towards the forward direction and, therefore, are not in accordance with PCI, which, for ionization of atoms and smaller molecules, usually shifts electron emission to the backwards direction. This might originate in the interaction of the outgoing electrons with the comparatively complex residual ion, which should be confirmed by future calculations. This set of experimental data will help to comprehensively evaluate the accuracy of theoretical models that account for the quantum few-body dynamics. Additionally, the absolute values of DDCS provide new input parameters for electron transport models based on Monte Carlo simulations. Finally, the semi-empirical formula we commonly used to obtain the inelastic angular distribution function for such models has been improved by extending its accuracy to higher energy transferred values, where the agreement with experimental data is highly satisfactory.

## Figures and Tables

**Table 1:** Ionization cross sections for the outermost MOs of benzene as calculated with the BEB method. The input is available online (https://physics.nist.gov).

MO	IE (eV)	σ_BEB_ (10^−20^ m^2^)
1e_1g_	9.25	3.93
3e_2g_	11.50	2.49
1a_2u_	12.15	1.34
3e_1u_	13.85	1.96
1b_2u_	14.63	0.89
2b_1u_	15.40	0.83
3a_1g_	16.85	0.76
2e_2g_	18.60	1.10
2e_1u_	22.10	0.56
2a_1g_	28.70	0.22

**Table 2:** Absolute DDCS for the production of the benzene parent ion C_6_H_6_^+^ at the impact energy of 90 eV for different transferred electron energies together with their estimated uncertainties (units are 10^−20^ m^2^ sr^−1^).

	30 eV		40 eV		50 eV	
Angle (deg)	DDCS	δ	DDCS	δ	DDCS	δ
4	-	-	0.1191	0.0238	-	-
7	0.2193	0.0438	0.0614	0.0123	-	-
10	0.1647	0.0329	0.0513	0.0102	-	-
13	0.1188	0.0237	0.0386	0.0077	-	-
16	0.0814	0.0163	0.0262	0.0052	0.0112	0.0022
19	0.0534	0.0107	0.02	0.0040	0.0095	0.0019
22	0.0383	0.0077	0.0161	0.0032	0.0079	0.0016
25	0.0294	0.0059	0.0131	0.0026	0.007	0.0014
28	-	-	0.011	0.0022	0.0062	0.0012
31	-	-	0.0107	0.0021	0.0054	0.0011
34	-	-	-	-	0.0046	0.0009
37	-	-	-	-	0.0042	0.0008
40	-	-	-	-	0.0036	0.0007
43	-	-	0.0055	0.0011	0.0034	0.0007
46	-	-	0.0047	0.0009	0.0035	0.0007
49	-	-	0.0035	0.0007	-	-
52	0.0046	0.0009	0.004	0.0008	-	-
55	0.0033	0.0007	-	-	0.0039	0.0008
58	-	-	0.0048	0.0010	0.0029	0.0006
75	-	-	-	-	0.0039	0.0008
78	-	-	0.0054	0.0011	-	-
81	-	-	0.0068	0.0014	0.0034	0.0007
96	0.0023	0.0005	0.0039	0.0008	0.0026	0.0005
99	0.0022	0.0004	-	-	-	-
102	-	-	0.0036	0.0007	0.0022	0.0004
117	-	-	-	-	0.0029	0.0006

## Data Availability

Not applicable.

## Abbreviations

DDCS	Double differential cross section
TDCS	Triple differential cross section
MO	Molecular orbital
HOMO	Highest occupied molecular orbital
IAM-SCAR+I	Independent atom model with screening corrected additivity rule and interference effects
BEB	Binary-encounter-Bethe method
PCI	Post collision interaction

## References

[1] García Gómez-Tejedor G., Fuss M.C., García Gómez-Tejedor G., Fuss M.C. (2012). Biological and Medical Physics, Biomedical Engineering. Radiation Damage in Biomolecular Systems.

[2] Boudaïffa B., Cloutier P., Hunting D., Huels M.A., Sanche L. (2000). Resonant formation of DNA strand breaks by low-energy (3 to 20 eV) electrons. Science.

[3] Sanche L. (2005). Low energy electron-driven damage in biomolecules. Eur. Phys. J. D.

[4] Pimblott S.M., LaVerne J.A. (2007). Production of low-energy electrons by ionizing radiation. Radiat. Phys. Chem..

[5] Blanco F., Muñoz A., Almeida D., Da Silva F.F., Limão-Vieira P., Fuss M.C., Sanz A.G., García G. (2013). Modelling low energy electron and positron tracks in biologically relevant media. Eur. Phys. J. D.

[6] Sanz A.G., Fuss M.C., Muñoz A., Blanco F., Limão-Vieira P., Brunger M.J., Buckman S.J., García G. (2012). Modelling low energy electron and positron tracks for biomedical applications. Int. J. Radiat. Biol..

[7] Costa F., Álvarez L., Lozano A.I., Blanco F., Oller J.C., Muñoz A., Barbosa A.S., Bettega M.H.F., Ferreira da Silva F., Limão-Vieira P. (2019). Experimental and theoretical analysis for total electron scattering cross sections of benzene. J. Chem. Phys..

[8] De Souza G.L.C., Dos Santos A.S., Lucchese R.R., Machado L.E., Brescansin L.M., Manini H.V., Iga I., Lee M.T. (2012). Theoretical investigation on electron scattering by benzene in the intermediate-energy range. Chem. Phys..

[9] Barbosa A.S., Bettega M.H.F. (2017). Shape resonances, virtual state, and Ramsauer-Townsend minimum in the low-energy electron collisions with benzene. J. Chem. Phys..

[10] Makochekanwa C., Sueoka O., Kimura M. (2003). Comparative study of electron and positron scattering from benzene (C6H6) and hexafluorobenzene (C6F6) molecules. Phys. Rev. A.

[11] Gianturco F.A., Lucchese R.R. (2017). One-electron resonances and computed cross sections in electron scattering from the benzene molecule One-electron resonances and computed cross sections in electron scattering from the benzene molecule. J. Chem. Phys..

[12] Itakura R., Watanabe J., Hishikawa A., Yamanouchi K. (2001). Ionization and fragmentation dynamics of benzene in intense laser fields by tandem mass spectroscopy. J. Chem. Phys..

[13] Kjeldsen T.K., Bisgaard C.Z., Madsen L.B., Stapelfeldt H. (2005). Influence of molecular symmetry on strong-field ionization: Studies on ethylene, benzene, fluorobenzene, and chlorofluorobenzene. Phys. Rev. A.

[14] Winney A.H., Lee S.K., Lin Y.F., Liao Q., Adhikari P., Basnayake G., Schlegel H.B., Li W. (2017). Attosecond Electron Correlation Dynamics in Double Ionization of Benzene Probed with Two-Electron Angular Streaking. Phys. Rev. Lett..

[15] Horio T., Maruyama R., Kishimoto N., Ohno K. (2004). A crossed-molecular beam study on collisional ionization dynamics of acetonitrile and benzene molecules with He*(23S) metastable atoms. Chem. Phys. Lett..

[16] Colyer C.J., Bellm S.M., Hanne G.F., Al-Hagan O., Madison D., Ning C.G., Lohmann B. (2011). Dynamical (e, 2e) studies using tetrahydrofuran as a DNA analogue. J. Phys. Conf. Ser..

[17] Builth-Williams J.D., Bellm S.M., Jones D.B., Chaluvadi H., Madison D.H., Ning C.G., Lohmann B., Brunger M.J. (2012). Experimental and theoretical investigation of the triple differential cross section for electron impact ionization of pyrimidine molecules. J. Chem. Phys..

[18] Dal Cappello C., Charpentier I., Houamer S., Hervieux P.A., Ruiz-Lopez M.F., Mansouri A., Roy A.C. (2012). Triple-differential cross sections for the ionization of thymine by electrons and positrons. J. Phys. B At. Mol. Opt. Phys..

[19] Khelladi M.F., Mansouri A., Cappello C.D., Charpentier I., Hervieux P.A., Ruiz-Lopez M.F., Roy A.C. (2016). Angular distributions in the double ionization of DNA bases by electron impact. J. Phys. B.

[20] Costa F., Traoré-Dubuis A., Álvarez L., Lozano A.I., Ren X., Dorn A., Limão-Vieira P., Blanco F., Oller J.C., Muñoz A. (2020). A Complete Cross Section Data Set for Electron Scattering by Pyridine: Modelling Electron Transport in the Energy Range 0–100 eV. Int. J. Mol. Sci..

[21] Dürr M., Dimopoulou C., Dorn A., Najjari B., Bray I., Fursa D.V., Chen Z., Madison D.H., Bartschat K., Ullrich J. (2006). Single ionization of helium by 102 eV electron impact: Three-dimensional images for electron emission. J. Phys. B.

[22] Ullrich J., Moshammer R., Dorn A., Dörner R., Schmidt L.P.H., Schmidt-Böcking H. (2003). Recoil-ion and electron momentum spectroscopy: Reaction-microscopes. Rep. Prog. Phys..

[23] Fuss M.C., Sanz A.G., Muñoz A., Do T.P.D., Nixon K., Brunger M.J., Hubin-Franskin M.-J., Oller J.C., Blanco F., García G. (2013). Interaction model for electron scattering from ethylene in the energy range 1–10000 eV. Chem. Phys. Lett..

[24] Potts A.W., Price W.C., Streets D.G., Williams T.A. (1972). Photoelectron spectra of benzene and some fluorobenzenes. Gen. Discuss. Faraday Soc..

[25] Stone P.M., Kim Y.-K. (2005). An overview of the BEB method for electron-impact ionization of atoms and molecules. Surf. Interface Anal..

[26] Karlsson L., Mattsson L., Jadrny R., Bergmark T., Siegbahn K. (1976). Valence electron spectra of benzene and the hexafluorides of sulphur, molybdenum, tungsten and uranium: An application of multichannel detector technique to UV-valence electron spectroscopy. Phys. Scr..

[27] Ren X., Amami S., Hossen K., Ali E., Ning C., Colgan J., Madison D., Dorn A. (2017). Electron-impact ionization of H2O at low projectile energy: Internirmalized triple-differential cross sections in three-dimensional kinematics. Phys. Rev. A.

[28] Hossen K., Ren X., Wang E., Gong M., Li X., Zhang S.B., Chen X., Dorn A. (2018). Triple-differential cross sections for single ionization of CO 2 by 100 eV electron impact. J. Phys. B.

[29] Ali E., Ren X., Dorn A., Ning C., Colgan J., Madison D. (2016). Experimental and theoretical triple-differential cross sections for tetrahydrofuran ionized by low-energy 26-eV-electron impact. Phys. Rev. A.

[30] Samardzic O., Brunger M.J., Grisogono A.-M., Weigold E. (1993). Electron momentum spectroscopy studies on ring compounds. I. Benzene. J. Phys. B.

[31] Wang E., Ren X., Gong M., Ali E., Wang Z., Ma C., Madison D., Chen X., Dorn A. (2020). Triple-differential cross sections for (e, 2e) electron-impact ionization dynamics of tetrahydrofuran at low projectile energy. Phys. Rev. A.

[32] Builth-Williams J.D., da Silva G.B., Chiari L., Jones D.B., Chaluvadi H., Madison D.H., Brunger M.J. (2014). Dynamical (e,2e) studies of tetrahydropyran and 1,4-dioxane. J. Chem. Phys..

[33] Jones D.B., Ali E., Ning C.G., Ferreira da Silva F., Ingólfsson O., Lopes M.C.A., Chakraborty H.S., Madison D.H., Brunger M.J. (2019). A dynamical (e,2e) investigation into the ionization of the outermost orbitals of R -carvone. J. Chem. Phys..

[34] Da Silva G.B., Neves R.F.C., Chiari L., Jones D.B., Ali E., Madison D.H., Ning C.G., Nixon K.L., Lopes M.C.A., Brunger M.J. (2014). Triply differential (e,2e) studies of phenol. J. Chem. Phys..

[35] Lozano A.I., Oller J.C., Jones D.B., Bettega M.H.F., Lima P., Lima M.A.P., White R.D., Brunger M.J., Blanco F., Mun A. (2018). Total electron scattering cross sections from para -benzoquinone in the energy range 1–200 eV. Phys. Chem. Chem. Phys..

[36] Blanco F., Rosado J., Illana A., García G. (2010). Comparison of two screening corrections to the additivity rule for the calculation of electron scattering from polyatomic molecules. Phys. Lett. Sect. A Gen. At. Solid State Phys..

[37] Blanco F., García G. (2004). Screening corrections for calculation of electron scattering differential cross sections from polyatomic molecules. Phys. Lett..

